# Dendritic Cell Subsets in Melanoma: Pathophysiology, Clinical Prognosis and Therapeutic Exploitation

**DOI:** 10.3390/cancers15082206

**Published:** 2023-04-08

**Authors:** Eleonora Sosa Cuevas, Philippe Saas, Caroline Aspord

**Affiliations:** 1 EFS AuRA, R&D Laboratory, 38000 Grenoble, France; eleonorasosa@hotmail.com (E.S.C.); philippe.saas@efs.sante.fr (P.S.); 2 Inserm U 1209, CNRS UMR 5309, Institute for Advanced Biosciences, Team: Epigenetics, Immunity, Metabolism, Cell Signaling and Cancer, Université Grenoble Alpes, 38000 Grenoble, France

**Keywords:** DC subsets, melanoma, immune subversion, prognostic factor, DC-based therapies

## Abstract

**Simple Summary:**

The clinical success of immunotherapies using immune checkpoint blockers deployed in melanoma strongly supports that the immune system can efficiently control tumor development in the long term. Yet, despite unprecedented successes, many patients still experienced relapse or failed to respond, highlighting the need to further explore the immune system interactions with tumor cells to develop efficient immunotherapeutic strategies. Since their discovery barely 50 years ago, dendritic cells (DCs) emerged as central regulators of immune responses. The DCs are active players in orchestrating anti-tumor responses but remain enigmatic as they harbor both anti-tumor and pro-tumor functions. This review aims to give an overview of the diversity of DC subsets, decipher their pathophysiology in melanoma patients and their impact on clinical outcome, the mechanisms by which tumors hijack DCs, and of their exploitation for therapeutic developments. Altogether, DCs hold great promise to participate in achieving better clinical outcomes for the patients in the future.

**Abstract:**

Evasion from immunity is a hallmark of cancer development. Dendritic cells (DCs) are strategic immune cells shaping anti-tumor immune responses, but tumor cells exploit DC versatility to subvert their functions. Unveiling the puzzling role of DCs in the control of tumor development and mechanisms of tumor-induced DC hijacking is critical to optimize current therapies and to design future efficient immunotherapies for melanoma. Dendritic cells, crucially positioned at the center of anti-tumor immunity, represent attractive targets to develop new therapeutic approaches. Harnessing the potencies of each DC subset to trigger appropriate immune responses while avoiding their subversion is a challenging yet promising step to achieve tumor immune control. This review focuses on advances regarding the diversity of DC subsets, their pathophysiology and impact on clinical outcome in melanoma patients. We provide insights into the regulation mechanisms of DCs by the tumor, and overview DC-based therapeutic developments for melanoma. Further insights into DCs’ diversity, features, networking, regulation and shaping by the tumor microenvironment will allow designing novel effective cancer therapies. The DCs deserve to be positioned in the current melanoma immunotherapeutic landscape. Recent discoveries strongly motivate exploitation of the exceptional potential of DCs to drive robust anti-tumor immunity, offering promising tracks for clinical successes.

## 1. Introduction

Tumor escape from the immune system’s control constitutes a hallmark of cancer development [1]. Recent strategies targeting the immune system (known as immunotherapies) are looking to restore anti-tumor immunity—by blocking tumor’s suppressive effects—to eradicate tumor growth. A high infiltration of several immune cell types in the tumors of melanoma patients has been linked with a better prognosis and a response to immunotherapies [2].

Dendritic cells (DCs)—discovered by Ralph Steinman 50 years ago—have emerged as central regulators of immune responses. Dendritic cells are essential for the initiation of immune responses by ensuring the link between innate and adaptive immunity. Dendritic cells are antigen-presenting cells specialized in antigen uptake and presentation or cross-presentation through their major histocompatibility complexes (MHC) type I and II [3]. They recognize different pathogen- or damage-associated molecular patterns (PAMPs or DAMPs, respectively) through their large panel of pattern recognition receptors (PRR), such as Toll-like receptors (TLRs), NOD-like receptors (NLRs), RIG-I-like receptors (RLRs) and lectins such as C-type lectin receptors (CLRs). Antigen recognition by PRRs induces different signaling pathways in DCs, responsible for DC maturation, activation and the regulation of their functions (cytokine production, phagocytosis) [4]. Dendritic cells play a pivotal role in tumor control via the uptake and presentation of tumor antigens, followed by their migration to lymph nodes, and the DC-dependent activation of naïve tumor antigen-specific CD4 or CD8 T-cells [5]. The DC responses are dependent on their environment—given their plasticity—and this can influence the orientation of the subsequent adaptive immune responses.

The adaptive response induced after antigen recognition can also vary depending on the DC subset presenting the antigen. Interestingly, differentiated DC subsets are present in humans and can be distinguished by their localization, cytokine production, migratory and antigen presentation capacities, and their expression of surface markers or PRRs [6]. These differences render them complementary and capable of responding to a large panel of stimuli. Furthermore—given their phenotypic and functional characteristics—each DC subset plays a specific role in anti-tumor immune responses. However, tumors can hijack DCs—by subverting their functions—and thus manipulate the immune system in their favor [5].

In this review, we will extensively describe the different human DC subsets present at basal state and during inflammation, their anti-tumor activity, as well as their subversion in the context of melanoma, the effect of current melanoma therapies on DC activity, and their exploitation for the development of novel therapeutic strategies targeting melanoma.

## 2. Different DC Subsets at Basal State and during Inflammation

### 2.1. Main Peripheral DC Subsets

Dendritic cells found in the blood are divided into three distinct main subsets: conventional DC type I or II (cDC1s or cDC2s, respectively) and plasmacytoid DC (pDCs). In humans, cDC1s—corresponding to CD8α^+^ DCs in mice [7]—represent less than 0.1% of peripheral blood mononuclear cells (PBMC) [8]. Their development is dependent on several transcription factors, such as IRF8 and BATF3 [9]. The cDC1s are distinguished from other DC subsets by their expression of BDCA3/CD141, XCR1 and DNGR1/Clec9α [10], and their higher potential for cross-presentation through their MHC-I—allowing them to induce effective cytotoxic immune responses against tumor cells after the recognition of tumor antigens (derived from dying tumor cells) by CD8 T-cells [11]. Upon TLR-3 triggering and induction of the transcription factor IRF3, cDC1s can also produce type III IFN (IFNλ1/IL29 and IFNλ2/IL28A), rendering them important for antiviral and anti-tumor immune responses [9].

The cDC2s represent the main DC subset found in the blood and lymphoid organs, and express BDCA1/CD1c and receptors TLR4 and TLR8—allowing the recognition of a large variety of DAMPs and PAMPs. After antigen uptake, activated cDC2s can produce TNFα, IL-1β, IL-6 and IL-8, and are considered as the major producers of IL-12p70 [12]. The cDC2s are also specialized in antigen presentation to CD4 T-cells through their MHC-II and can orientate T-cell differentiation towards different profiles (Th1, Th2, Th17) depending on their environment. They are also capable of inducing CD8 T-cell responses, but to a lesser extent than cDC1s—given their limited capacity to cross-present antigens derived from necrotic cells [8,13].

On another note, pDCs are differentiated by their expression of BDCA2/CD303 and CD123 and are the main producers of type I IFN upon TLR-7/9 stimulation after ligation of DNA or RNA extracellular complexes [14,15]. The pDCs could also present antigens to T-cells and induce adaptive immunity. However, previous studies of pDCs could be biased by their contamination by a cDC precursor also expressing BDCA2 and CD123 (known as Axl^+^/SIGLEC-6^+^ [AS] AS-DCs) and capable of differentiating into cDCs [6,16,17]. Given the range of actions attributed to pDCs which could actually be due to contaminating AS-DCs, further studies of “pure” pDCs (Axl^−^BDCA2^+^CD123^+^)—excluding AS-DCs—should be done to assess pDC specific functions.

### 2.2. Other DC Subsets

#### 2.2.1. At the Basal State

Resident DCs can be found in human tissue such as the skin. Langerhans cells (LCs)—located in the epidermis—express Langerin/CD207 [18]. They can produce large amounts of IL-15. The LCs are also capable of inducing CD4 T-cell differentiation into Th2 cells and have a high capacity for cross-presentation—allowing them to efficiently activate naïve CD8 T-cells [19]. The CD1a^+^ or CD14^+^ DCs can also be found in the dermis. The CD14^+^ DCs are capable of inducing CD4 T-cell differentiation and B-cell activation [19], whereas the action of CD1a^+^ DCs is not well known even though they seem to be active given their high activation marker expression [20].

Recently, several studies have indicated a heterogeneity of some DC subsets (mostly cDC2s) in the periphery [6,21,22]. At first, following single cell RNA sequencing (scRNAseq) analyses, two human cDC2 subsets were characterized depending on CD14 and CD163 expression [6]. Other studies also distinguished two types of cDC2s given their CD5 expression [23], with high expression of SIGLEC-6 and IRF4 found in CD5^+^ cDC2s and high levels of CD14 and CD163 in CD5^lo^ cDC2s. These observations allowed the characterization of two subsets of cDC2s: CD5^lo^CD14^+^CD163^+^ cDC2s and CD5^hi^CD14^−^CD163^−^ cDC2s. However, Bourdely et al. identified CD5^lo^CD14^+^CD163^+^ cells as a distinct DC subset from cDC2s, called DC3s [24]. The DC3s were found in the blood at the steady state, expressed BDCA1, CD163, CD14, FcεRI and CD206, and differentiated from CD34^+^ progenitors upon GM-CSF stimulation—independently from cDC progenitors and monocytes. The DC3s were capable of inducing CD4 and CD8 T-cell activation, even though less efficiently than cDC2s, and could produce IL-12 and IL-23 [24]. In addition, single cell analyses revealed new subsets of cDC2s—named cDC2A and cDC2B—differentially expressing T-bet and RORγt, respectively, and harboring distinct metabolic and functional programs with pro- or anti-inflammatory potential [25]. cDC2A are CD1c^lo^CLEC10A^−^CLEC4A^hi^ and exhibited immunomodulatory features (AREG, IDO1, CD300a, IL22RA2), whereas cDC2B are CD1c^+^CLEC10A^+^CLEC4A^lo^, display a pro-inflammatory phenotype (IL-1β), and harbor genes associated with lipid antigen presentation and metabolism.

#### 2.2.2. During Inflammation

Following an infection or inflammation, monocytes were thought to differentiate into inflammatory DC (moDCs/InfDCs) [3]—expressing monocyte (CD14, CD16) and DC (MR/CD206, DC-SIGN/CD209) markers—capable of acting in situ at the inflammation site (low migratory capacity). The MoDCs could also be generated in vitro from monocytes in the presence of GM-CSF and IL-4, followed by stimulation with LPS. However, ex vivo culture of “pure” monocytes (CD88^+^CD14^+^) were unable to produce moDCs (CD88^−^CD14^+^CD1c^+^) after the appropriate stimuli [24], whereas DC3s differentiated into moDCs after GM-CSF stimulation. Further studies are thus necessary to define the origins of infDCs in vivo during inflammation (monocytes, DC3s or both).

In addition, Alculumbre et al. showed that pDCs derived from healthy donors can differentiate into three stable subsets—distinguished by their CD80 and PD-L1 expression and possessing functional specificities—after a single stimulus [26]. These pDC subsets can also be found in autoimmune diseases (lupus and psoriasis) [26], after viral infections (SARS-CoV-2) [27], and in the context of melanoma [28].

Furthermore, a subset of DCs harboring an inflammatory phenotype—called mregDCs—can be found in several cancers and in different settings of inflammation (psoriasis, Crohn disease), but absent in the blood of these patients [29]. The MregDCs show a downregulation of genes responsible for TLR signaling, an overexpression of immune-modulatory molecules (PD-L1, PD-L2, CD200) and of maturation and migration markers (CD40, CCR7, IL-12) [5]. Given mregDCs’ transcriptomic resemblance to cDC1s (XCR1, CD103) and cDC2s (CD11b), these cells would be originated from cDCs after the recognition of specific antigens. The analysis of mregDCs should be integrated in further studies of tumor-infiltrating DC subsets by using markers such as CCR7, LAMP3 and CD11c to distinguish them from other DC subsets.

The main characteristics and functions of the DC subsets described above—at the basal state and during inflammation—are summarized in Table 1.

### 2.3. Heterogeneity of Intratumoral DC Subsets

All DC subsets found in the blood and inflamed tissues have been reported to infiltrate tumors. These DC subsets including cDC1s, cDC2s, pDCs, but also LCs, DC3s, InfDCs and mregDCs harbor a large spectrum of antitumor and tolerogenic properties within the TME [5,30,32,33] that crucially orchestrate the subsequent antitumor responses. Crosstalk between these DC subsets within the tumor microenvironment may modulate the overall immune response (see Section 3) [34].

## 3. DC Subsets in Melanoma: Heterogeneity, Function, Subversion and Prognosis

### 3.1. Anti-Tumor Activity of DCs

**Figure 1:**
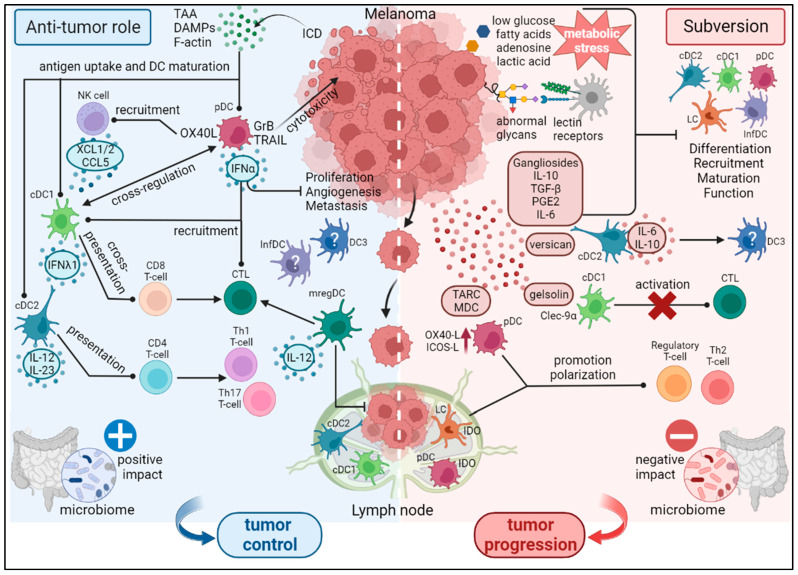
Dual role of DC subsets during melanoma development. DCs are central regulators of anti-melanoma immune responses and subsequent outcome. DCs display a puzzling role in the control of tumor development as they harbor both anti-tumor and pro-tumor functions. On one hand (left part), DCs are active players in orchestrating robust anti-tumor immunity by triggering cytotoxic CD8+ T cells through TAA cross-presentation, recruiting and activating NK cells, ending up in tumor control. On the other hand (right part), melanoma tumor cells exploit DC versatility to subvert their functions. Many factors secreted by melanoma tumor cells interfere with DC differentiation, maturation and function, leading to immunosuppressive/tolerogenic DCs and driving regulatory and Th2 responses. Abnormal glycans, unfavorable metabolic environment and specific microbiome can also negatively impact DC subsets. All these pathways favor tumor progression. This figure illustrates both the intra-tumoral heterogeneity of the DC subsets encountered in melanoma, the potential crosstalk between these DC subsets, and the prognostic value of DC subsets in melanoma.

Dendritic cell subsets were firstly identified in tumors by in situ immunohistochemistry (IHC) using antibodies targeting specific DC subset markers [30]. However—to overcome the limited number of markers visualized by IHC—multi-parametric flow cytometry panels were developed to study simultaneously several DC subsets. Furthermore, the development of high throughput technologies (CyTOF, scRNA-seq) also allowed the extensive characterization of previously known DC subsets and the identification of new DC subsets (like DC3s). In the context of melanoma, several DC subsets have been shown to infiltrate the tumors [28,35,36,37,38,39], and—given the different functions of DC subsets—DC heterogeneity in the immune infiltrate could affect subsequent anti-tumor immunity (Figure 1).

The anti-tumor role of cDC1s is mostly due to their higher capacity for cross-presentation—leading to the activation of cytotoxic CD8 T-cells—and their aptitude to induce Th1 polarization of CD4 T-cells after antigen presentation [40,41]. The recognition by cDC1s of DAMPs, tumor antigens—released after immunogenic cell death (ICD)—, and necrotic cells—through the recognition of actin filaments (F-actin) by Clec9α—induces maturation, cross-presentation, and subsequent activation of T-cells. Given their migratory capacity, cDC1s located in peripheral tissues can thus uptake tumor antigens and migrate to tumor-draining lymph nodes where they initiate adaptive immune responses. Furthermore, cDC1s’ type III IFN production plays an anti-tumor role by the induction of IL-12p70 and IFN-γ secretion in the tumor microenvironment (TME)—resulting in an antitumor Th1 microenvironment [42].

Recruitment of cDC1s into the tumor site is also crucial for their anti-tumor activity and depends on the presence of different chemokines in the TME—such as XCL1, XCL2 and CCL5—mainly produced by NK cells [43,44]. Interestingly, the study of TCGA datasets from several cancer types brings to light a correlation between cDC1 gene signature (*CLEC9A*, *XCR1*, *CLNK*, *BATF3*), cDC1 chemoattractants in the TME, and NK/CD8 T-cell gene signatures [44]. Furthermore, FLT3 ligand (Flt3L) production by NK cells enhances cDC1 proliferation and survival in the TME, whilst IFN-γ and TNFα secretion by NK cells increased tumor antigen cross-presentation by cDC1s [45].

As for cDC2s, the presentation of tumor antigens directly to CD4 T-cells—needed for CD8 T-cell activation—or indirectly by transferring tumor antigens to lymph node resident cDC2s represent the major roles of cDC2s in anti-tumor immunity [46]. In addition, the production of specific cytokines (IL-12p70, IL-23) by cDC2s can orientate Th1 or Th17 polarization of CD4 T-cells and induce the expansion of memory CD8 T-cells [12,47]. Vaccination with tumor-derived cDC2s induced the Th17 polarization of CD4 T-cells, reprogramming of pro-tumor macrophages, and a decrease of myeloid-derived suppressor cells (MDSC)—resulting in tumor size reduction in a tumor mice model [47]. Furthermore, cDC2s could facilitate the recruitment of NK cells into the TME—favoring their anti-tumor activity and possibly cDC1 recruitment [48]. Crosstalk between cDC could be possible through IL-12 production—needed for the efficient induction of Th1 responses by cDC2s—or via CD40L-expressing CD4 T-cells [34]. Notably, a recent study by the group of E. Klechevsky revealed the crucial role of CD5^+^CD1c^+^ cDC2s in driving anti-tumor immunity and sustaining immunotherapy responses [49].

Plasmacytoid DCs have also been studied in tumors and can favor anti-tumor immunity through the production of type I IFN, which plays a cytotoxic role through the inhibition of proliferation, angiogenesis and metastasis formation. Type I IFN can also induce de recruitment of cDC1s and cytotoxic T lymphocytes (CTLs) and the local production of type I IFN by pDCs in tumor mouse models can activate CD8 T-cells through cDC1s [50,51]. Intratumoral pDCs activated with imiquimod (TLR7/8 ligand) can induce a TRAIL- and granzyme B-dependent cytotoxic response against tumors, inhibiting their development [52,53].

The pDCs are also able to uptake and present tumor antigens to T-cells, even though less efficiently than cDCs [54,55,56]. The pDCs can process synthetic tumor-derived peptides (such as MelA) and present or cross-present antigens to CD4 or CD8 T-cells, respectively [54]. The pDCs activated and pulsed with tumor peptides—delivered to melanoma patients through intranodal injection—could migrate to the lymph nodes where they efficiently activated anti-tumor CD4 and CD8 T-cells [55]. In addition, the injection of activated pDCs into a B16 melanoma mice model induced the recruitment and activation of NK cells in the tumor site—in a dependent manner of OX40L-expressing pDCs [57].

Interestingly, by performing spatially resolved transcriptomic analysis of CD14^+^ cells from different localization in melanoma tumor sections, the group of K. Palucka identified a DC signature (CD14^+^CD2^+^LY75^+^) in the stroma linked with antigen capture and presentation. This signature is associated with long-term survival of melanoma patients [58]. This discovery suggests the potential reprogramming of stromal macrophages to acquire DC phenotype and function.

The MoDCs—derived in situ from tumor-infiltrating monocytes recruited by CSF-1 and CCL2—lack CCR7 expression and their anti-tumor activity would mostly entail an in situ activation of T-cell cytotoxicity in tumors. Moreover, higher levels of moDCs are found in immunotherapy responder compared to non-responder melanoma patients [59]. MoDCs could also originate from DC3s [24]—not yet identified in melanoma, but found in other cancer types (oropharyngeal and breast)—which can play an anti-tumor role by the production of IL-12p70 and IL-18, leading to the Th1 polarization of T-cells [60]. The presence of tumor-infiltrating DC3 was also positively correlated with the infiltration of Th1 T-cells [60] and the frequency of CD8^+^CD103^+^CD69^+^ resident memory T-cells [24]—associated with a better prognostic in breast cancer [61].

As for mregDCs—firstly described in melanoma by their expression of DC-LAMP/LAMP3—they were associated with the local expansion of memory cytotoxic T-cells and the absence of lymph node metastasis [62], hypothesizing an anti-tumor activity for mregDCs. Such LAMP3^+^ DCs have also been identified in the brain metastases of melanoma patients. In these patients, they have been associated with increased overall survival [63]. They displayed a strong correlation with infiltration by CD8 T-cells, suggesting that they positively regulate the immune environment. Following IFN-γ production by NK or T-cells, mregDCs can produce IL-12 and stimulate anti-tumor immunity (CD4 or CD8 T-cells, NK cells) [64]. However—depending on their origin (cDC2s or cDC1s)—mregDCs can have different actions. The expression of IL-12B and BTLA is exclusive to cDC1-derived mregDCs, whereas CD1E and CCL17 are only expressed by cDC2-derived mregDCs [29].

### 3.2. Subversion of DCs within the TME

Despite exhibiting potent anti-tumor properties, DCs were found to be hijacked by melanoma tumors [65,66] (Figure 1).

The cDC1s were found diminished in the circulation and present in melanoma tumors, however this subset remains scarce [37,67]. Studies of cDC1 in human tumors are limited and subversion mechanisms have been mostly described using mouse models. The activity of cDC1 seems to be mostly regulated by their exclusion from the tumor site, and not by a direct effect of the tumor on their activity. The production of PGE2 by the tumor affects the NK-cDC1 axis and leads to the exclusion of cDC1 from the tumor site in a mouse model [44], emphasizing the importance of this axis for the recruitment of cDC1s and their subsequent anti-tumor activity. In a melanoma mouse model, the decrease of CCL4 secretion by tumor cells—following the activation of the β-catenin/Wnt pathway—inhibits cDC1 recruitment into the tumor site and leads to a decrease of the subsequent T-cell infiltration [68]. Tumors can also modulate cDC1s’ cross-presentation capacity of tumor antigens—through gelsolin secretion in the TME of a melanoma mouse model—by inhibiting F-actin recognition by Clec9α, leading to a decreased proportion of tumor-infiltrating cytotoxic T-cells [69]. However, in melanoma patients, circulating and tumor-infiltrating cDC1 preserve a potent functionality and could be reactivated ex vivo using TLR ligands, which induce the production of IFNλ1 and TNFα in levels comparable to controls [39]. This suggests that cDC1s could escape from tumor-induced immune subversion, and their potent capacities represent a driving force to trigger protective anti-tumor immune responses.

In contrast to cDC1s, cDC2s and pDCs display defective functions in melanoma. The impact of melanoma cells on cDC2s’ anti-tumor potential has mostly been studied using mouse models [46]. In a B16 melanoma mouse model, CD11b^+^ DCs—the homolog of cDC2s in humans—infiltrate the tumor site but display a reduced antigen presentation capacity through MHC-II, causing a weak activation of CD4^+^ T-cells in lymph nodes [70]. Further study of CD11b^+^ DCs from lymph nodes demonstrate that they have the potential to present tumor antigens to CD4 T-cells but without inducing their differentiation, due to the presence of Treg [71]. Indeed, Treg depletion improves the capacity of CD11b^+^ DCs to trigger CD4 T-cell responses, leading to a better control of the tumor. In addition, in vivo in an advanced melanoma model, tumor-infiltrating CD11b^+^ DCs do not migrate to the lymph nodes [72]. It has been demonstrated that CD11b^+^ DCs uptake lysosomes present in tumor-secreted exosomes leading to their death. The absence of CD11b^+^ DCs prevents the anti-tumor cytotoxic activity of T lymphocytes. Furthermore, tumor-derived exosomes are able to trigger in vitro the death of CD11b^+^ DCs derived from healthy donors [72]. The analogue of this subtype of DCs—cDC2s—has been found in patients in the tumor draining lymph nodes. Furthermore, in a mouse B16 melanoma model, the differentiation of cDC precursors has been shown to be modulated by the production of TLR2 ligands by the tumor [73]. Indeed, tumors produce the TLR2 ligand, versicane that leads to a dysfunctional activity of cDC2s driving the production of IL-6 and IL-10. Blocking TLR2 in vivo improved DC response and increased the efficacy of immunotherapies. Interestingly, a signature of the newly described cDC2A subset displaying immunomodulatory features (AREG, NR4A3) has been found in human melanoma tumors [25].

Moreover, pDCs found in invaded lymph nodes appear to have a partial maturation and lower capacities to produce IFNα [74]. In tumors, the recruitment of pDCs would occur through the CCR6-CCL20 [75] and CXCR4-SDF1 [76] axes. Indeed, the production of CCL20 by the tumor recruits circulating pDCs that express higher levels of CCR6 compared to pDCs of healthy donors. In addition, the production of IFNα by pDCs can be impacted by soluble factors such as PGE2, IL-10 and TGFβ [77], lactic acidosis [78] and by the engagement of LAG3 which leads to the activation of tolerogenic pDCs [79]. Circulating pDCs expressing LAG3 with a reduced production of IFNα and an increase in IL-6, are also present in patients’ tumors. Other products derived from the tumor, such as adenosine or factors secreted via the Wnt5a pathway can also impact the activity of pDCs. Indeed, Wnt5a signaling in tumors blocks the expression of co-stimulation molecules (CD80, CD86) and the production of IFNα by pDCs after stimulation with a ligand of TLR9 (CpG), leading to the presence of dysfunctional pDCs in the TME [80]. In addition, the recognition of adenosine by mature human pDCs induces an increase in cytosolic cAMP and an inhibition of their cytokine production (IFNα, IL-6) [81]. Tumor-infiltrating pDCs also display a high expression of OX40L and ICOSL allowing them to promote T-cell profiles (Th2, Treg), which can support tumor progression [36]. In addition, the presence of TARC/CCL17, MDC/CCL22 and MMP-2 in the tumor microenvironment was correlated with the modulation of OX40L and ICOSL in pDCs and their accumulation in the tumor.

The interaction between pDCs and other anti-tumor immune actors can also be impacted in melanoma. The activity of γδ T-cells is strongly disturbed in melanoma [82], following the inability of intratumoral pDCs to activate them and modulate their expression of immune checkpoints [83]. Furthermore, in a mouse model, tumor-infiltrating pDCs express IDO and can regulate anti-tumor T-cell responses and induce an immunosuppressive environment [84]. As IDO expression was also found in more than half of the pDCs present in the sentinel lymph nodes of patients, the results observed in mice suggest a tolerogenic action of IDO expression by human pDCs [85].

Langerhans cells (LCs) are found in the sentinel lymph nodes of melanoma patients, where their activation status appears to be altered [86] with a low ability to induce allo-reactive T-cells [87]. Langerhans cells acquire mobility under the influence of TGF-β and promote immune suppression through Treg expansion. In addition, these LCs are not able to capture exogenous antigens but have a fully functional antigen-presenting machinery [88]. Recently, a subtype of tolerogenic LCs (IDO^+^CD83^−^) has been detected in a large proportion of sentinel lymph nodes with micrometastases compared to sentinel lymph nodes not infiltrated by tumor cells [89]. This subtype of LCs has also been found in the sentinel lymph nodes of patients whose primary tumor has an intermediate or strong mitotic index (greater than 1).

In an in vitro model of spheroids derived from melanoma tumor lines, the differentiation of monocytes into moDCs is impacted in the presence of spheroids [90]. Indeed, the lactic acid produced by tumors leads to the production of tolerogenic moDCs (CD1a^−^CD14^+^) with a decreased production of IL-12, IL-6 and TNFα. These cells are unable to prime CD8 T-cells. In addition—following the in vitro reconstitution of a human melanoma TME using tumor lines (BLM, Mel624 or A375)—PGE2 and IL-6 produced by the tumor transform cDC2s into CD14^+^ DCs, characterized by an immunosuppressive phenotype [91]. Indeed, tumor-infiltrating CD14^+^ DCs express high levels of CD206 and CD163 and could correspond to moDCs and/or DC3s [24]. In addition, CD14^+^ DCs are found in the tumors of melanoma patients and express high levels of PD-L1, allowing the inhibition of T-cell proliferation [91,92].

Inter-DC cross-talks are crucial for effective anti-tumor immune responses [34]. Yet, melanoma drastically tuned interrelations between DC subsets. Indeed, perturbed inter-relations between DC subsets were observed in both blood and tumor of melanoma patients regarding the frequency, the basal activation status, and the response to TLR triggering [39]. Positive interactions between cDC2s, pDCs, and cDC1s are lost in both blood and tumor of melanoma patients, whereas new interactions emerged within tumor microenvironment especially between pDCs and cDC1s. Such perturbations may lead to melanoma escape from the immune control. Inter-DC synergistic cooperation is crucial for efficient cross-priming of anti-tumor responses and should be considered for the development of optimal DC-based immunotherapies to achieve robust anti-tumor immunity and maximal clinical success.

### 3.3. Factors Influencing DC Function in Melanoma

#### 3.3.1. Melanoma-Derived Factors

The DAMPs released by dying tumor cells act as danger signals for DCs, and promote their maturation, activation and subsequent ability to stimulate immune responses [93]. For example, surface calreticulin (CRT), secretion of ATP and IL-1β, or release of HMG-B1 and nucleic acids drive DC modulation following interaction with the sensors such as RIG-I, TLR4, P2X or CD91. Such DAMPs also promote type I IFN secretion, and further trigger CXCL10 secretion that promotes the recruitment of immune effectors to the tumor. Thus, inducers of ICD—such as chemotherapies—have the potential to promote DC-mediated anti-tumor immune responses.

On the opposite, a variety of factors present in the TME alter DC function by interfering with DC differentiation, maturation, activation and function [94]. The COX-derived prostanoids and gangliosides produced by melanoma tumor cells inhibit the differentiation of DCs from monocytes and CD34^+^ progenitors and trigger their apoptosis [95]. The TGF-β1 and PGE2 secreted by melanoma cells were shown to skew the differentiation of DC precursors towards myeloid populations with immunosuppressive function such as MDSCs [96]. The IL-10 secreted in high levels by melanoma promote the trans-differentiation of moDCs into tolerogenic CD14^+^ BDCA3^+^ macrophage-like cells similar to the one enriched in melanoma metastases [97]. These melanoma-derived factors also suppress costimulatory molecule expression and cytokine secretion by DCs, and trigger regulatory DCs with tumor-promoting functions through the driving of tumor angiogenesis, the recruitment of immunosuppressive Treg, and the suppression of T-cell responses [93].

#### 3.3.2. Tumor Glycocode

In addition to soluble factors and regulatory molecules found in the TME, hijacking of DCs could be triggered by aberrant glycosylation patterns present on the tumor cell surface. It has been documented that melanoma tumor cells exhibit a high ganglioside diversity, display alterations in the glycosylation pattern of glycoproteins and glycolipids, and major perturbations of the expression of enzymes involved in glycosylation/deglycosylation processes (glycosyl-transferases, glycosidases) [98,99]. Strikingly, altered carbohydrate patterns on tumor cells can be recognized by glycan-binding receptors, such as C-type lectin receptors (CLR), especially expressed by DCs. Emerging evidence suggest in a few cancer types an immunosuppressive role of altered tumor glycosylation on innate immune responses through modulation of immune cells’ function [100]. In melanoma patients, we recently highlighted that the CLR profiles in circulating and tumor-infiltrating DC subsets display strong perturbations, correlated with unique DCs’ features, and dictated clinical outcomes [101]. Furthermore, the melanoma tumor glycocode directly affects the DC subsets’ functionality [102], revealing the glycan/CLR axis as a potential DC subversion pathway in melanoma.

#### 3.3.3. Impact of the Metabolism on DCs’ Function

The emerging role of immunometabolism in the regulation of DC function revealed new mechanisms of tumor-induced DC subversion. Tumor cells undergo a metabolic switch from OXPHOS to glycolysis to support active proliferation, creating an environment hostile to infiltrating DCs, with the competition for limiting nutrients and accumulation of toxic metabolic products in the TME that impair immune cell function [93,103]. Depletion of glucose in the TME by melanoma cells impair glycolysis and subsequent ATP production by tumor-infiltrating DCs, whereas accumulation of lactic acid inhibits DC differentiation and suppresses DC function [78]. The accumulation of lipids in tumor-associated DCs impaired antigen processing and cross-presentation, and fatty acids inhibit DC maturation. Furthermore, adenosine, which accumulates in melanoma TME upon hydrolysis of ATP by CD39 and CD73 ectonucleotidases, has been shown to impair DC function through A_2A_ adenosine receptor [104]. Thus, metabolic reprogramming of DCs by tumor cells contributes to DC dysfunction and tumor immune escape in melanoma.

#### 3.3.4. Role of the Microbiome in Shaping DC Immunogenicity

Much evidence suggest that the gut microbiome influences the quality of anti-tumor immunity in melanoma [93]. Alteration of the composition of the microbiota affected both natural and therapy-associated anti-tumor responses. It has been suggested to be linked with microbial influence on DC activation. In a mouse model of melanoma, antibiotic treatment prior tumor challenge enhanced tumor growth and is associated with decrease DC infiltration, maturation, activation, and impaired immune control [105]. Notably, the microbiome has been shown to affect DC function and anti-tumor immunity in the context of immune checkpoint blocker (ICB) therapies in melanoma. Indeed, response to anti-PD-L1 therapy relies on the presence of specific intestinal *Bifidobacterium* species, and is associated with the frequency of tumor-infiltrating DCs, as well as signatures of genes involved in DC maturation, antigen presentation, and chemokine involved in the recruitment of effectors [106]. These insights support further developments to manipulate the gut microbiome to promote DC function and subsequent anti-tumor immunity in melanoma.

### 3.4. Prognostic Impact of DC Subsets in Melanoma

High levels of mature tumor-infiltrating DCs are inversely correlated with tumor progression and represent a favorable prognostic factor in various cancers including melanoma [107]. Frequencies of circulating and/or tumor-infiltrating DC subsets display perturbations in melanoma patients that drastically correlated with clinical outcome.

The cDC1s are very rare, yet their level strongly correlates with clinical prognosis and response to immunotherapies. The presence of a cDC1 transcriptomic signature in tumors of melanoma patients has been correlated with a better clinical outcome [43]. A higher proportion of circulating cDC1s [39], as well as a high infiltration of tumors by activated cDC1s [38] or IFN-λ1 secreting cDC1s [39] is correlated with a good prognosis. In a mouse model of melanoma, the presence of cDC1s within tumors was essential for the efficacy of immunotherapies [108,109]. The cDC1s also secrete different chemokines (CXCL9, CXCL10) required to attract cytotoxic lymphocytes into the tumor microenvironment, essential for the response to CAR T-cell therapy [110]. In addition, CCR7 expression—necessary for cDC1s migration—is correlated with a genetic signature of cDC1s, the presence of intratumoral T-cells and a better clinical response of melanoma patients [38]. Strong transcriptomic expression of specific markers of cDC1s is also correlated with better clinical prognosis in other types of cancers (breast cancer, HNSC, LUAD) [41,42]. The presence of key cDC1-specific genes (THBD, CLEC9A, XCR1) in tumors was correlated with a CD8 T-cell signature [42].

Some melanoma patients responding to immunotherapies also have high levels of intratumoral cDC1s and CD8 T-cells, while the other responders have high proportions of cDC2s and CD4 T-cells [71]. In addition, in tumors with a low proportion of Treg, intratumoral density of cDC2s was correlated with high conventional CD4 T-cell abundance and better response to anti-PD-1 [71]. Non-responding patients generally have low frequencies of tumor-infiltrating cDCs. In addition, high LDH levels are also correlated with a decrease in the proportion of cDCs and an increase in Treg in patients with stage IV melanoma, and the absence of cDCs after tumor excision was correlated with the risk of relapse [111].

Intratumoral abundance of pDCs is also correlated with a poor patient prognosis [36,77]. However, recent studies suggest that there is a decrease in the proportion of pDCs during melanoma progression despite their tolerogenic action [78,112]. Indeed, the exposure of CD34^+^ precursors of pDCs and of differentiated pDCs to soluble tumor components could respectively block their differentiation or induce their apoptosis and be at the origin of the decrease in the proportions of pDCs in circulation and in the metastases of patients. In addition, this reduction of pDCs in metastatic melanoma (especially in patients with a high LDH level) would be due to a high production of lactic acid by tumors that would reduce the survival and function of pDCs [78]. This reduction in pDCs has been associated with poor patient prognosis. By exploring pDC diversity, we observed an increased frequency of P3-pDCs (CD80^+^PD-L1^−^) in the blood of melanoma patients compared to healthy volunteers, together with their accumulation within the tumor, which was linked to a bad clinical outcome [28]. According to Alculumbre et al., P3-pDCs are well equipped to stimulate naive CD4 T-cells toward a Th2 profile [26]. Thus, the phenotypic and functional heterogeneity of pDCs in melanoma patients may shape clinical outcomes.

Several studies relative to other specific observations also highlighted the prognostic value of other DC or DC-related subsets in melanoma. The presence of mregDCs, associated with the presence of activated T-cells, constitutes an independent prognostic factor linked to a functional anti-tumor response [107]. Such LAMP3^+^ DCs, identified in the brain metastases of melanoma patients, have been associated with increased overall survival [63]. Indeed, an increase in the genetic profile associated with mregDCs is linked to the presence of tertiary lymphoid structures (TLS) in tumors, and to a better prognosis of patients in several cancers (breast, NSCLC, CRC) including melanoma [5]. Moreover, a DC signature (CD14^+^CD2^+^LY75^+^) associated with stromal macrophages linked with antigen capture and presentation was found associated with long-term survival of melanoma patients [58].

## 4. Effects of Current Melanoma Therapies on DC Subsets’ Features

**Figure 2:**
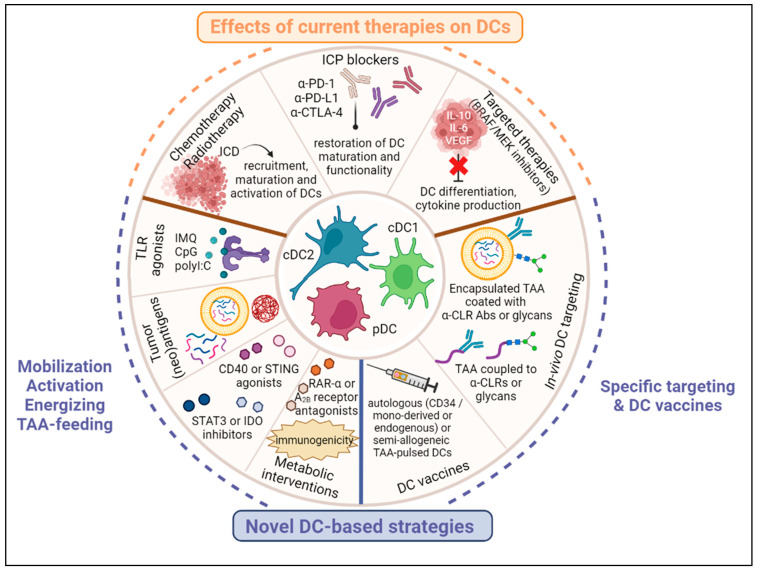
DC-based immunotherapeutic strategies in melanoma. Current therapies such as chemotherapies, immune checkpoint blockers and targeted therapies deployed in melanoma can indirectly positively affect DCs’ recruitment, differentiation, maturation and function. Besides, insights into the mechanisms of DCs’ hijacking by melanoma led to the design of therapeutic strategies that exploit the potential of DCs while preventing their subversion by the tumor. These strategies include approaches that mobilize, activate, energize and feed endogenous DCs, interventions that target specific DC subsets in vivo using TAA coupled to anti-CLR Abs or glycans, and DC vaccines.

Some already existing therapies can indirectly affect the properties of DCs, by promoting their immunogenic functions and restoring some of their functions that have been blocked by the tumor (Figure 2). Several reviews have deciphered in detail the impact of current anti-tumor therapies on immune cells [31,113,114]. We described below knowledge regarding such impact on DCs and specifically in melanoma.

### 4.1. Chemotherapy and Radiotherapy

In addition to direct cytotoxic activity toward tumor cells, chemotherapies participate in the restoration of the subverted properties of DCs by the induction of ICD of tumor cells [115]. Following the release of alarmins (e.g., ATP, HMGB1) and exposure of calreticulin, ICD induces the recruitment, maturation, and activation of DCs in TME, and favors cross-presentation of tumor-associated antigens (TAA). Such positive impact of chemotherapies on anti-tumor responses has been proven to be dependent on DCs in several preclinical melanoma mouse models [116]. Dacarbazine, a chemotherapeutic agent used for the treatment of melanoma, promotes the efficacy of a peptide-based vaccine in patients by enhancing the T-cell receptor repertoire diversity of antigen-specific CTL, suggesting an ICD-mediated benefit [117].

Radiotherapy can also induce ICD and DNA release from the tumor, acting as DAMPs capable of inducing the production of type I IFN by DCs. However, at low doses, radiotherapy can induce the expression of TREX1, capable of degrading DNA in the cytosol and limiting the activity of DCs [118].

### 4.2. Targeted Therapies

The blockade of MAPK signaling using BRAF/MEK inhibitors has been shown to reduce the production of IL-10, IL-6, and VEGF by melanoma cells, and to inhibit their negative effect on DC differentiation, cytokine production and antigen cross-priming, thus restoring their functions and subsequent anti-melanoma immunity [65,119].

### 4.3. Immunotherapies

Immunotherapies (anti-PD-1, -PD-L1, -CTLA-4 monoclonal antibodies) can also impact DCs and restore their maturation and functionality (i.e., cytokine production, priming of cytotoxic lymphocytes) [31]. In addition, in mouse models of melanoma, cDC1s appear to play an important role in the response to immunotherapies targeting PD-1 [108,109]. The reactivation of intratumoral cDC1s by administration of Flt3L could render tumor cells sensitive to ICBs [120]. Considering the importance of DCs for the response to ICBs, these treatments can be combined with DC activators (Flt3L, cGAMP, polyI:C, anti-CD40 antibodies) to improve the response to treatment. It is nevertheless interesting to note that the administration of Flt3L in melanoma patients can also induce Treg expansion, thus promoting the development of an immunosuppressive environment [121]. Administration of Flt3L is not always favorable for anti-tumor responses.

## 5. Harnessing DC Subsets for Therapeutic Strategies in Melanoma (Figure 2)

### 5.1. In Vivo Activation, Mobilization and TAA-Feeding of DCs

Due to the potential of DCs to initiate the control of tumor progression, therapeutic strategies in development seek to target these cells by favoring their recruitment, survival, and reactivation in the TME while potentiating their functions [115].

The in vivo delivery of TAAs that can be presented by DCs is an attractive approach to facilitate antigen uptake by DCs present in tumors and to induce antigen-specific anti-tumor responses [115]. The efficacy of this type of treatment depends on cancer mutational load, allowing them to be distinguished from healthy cells and specifically targeted. The TAAs used can be of different forms (short or long synthetic peptides, tumor lysate, recombinant viruses expressing the tumor antigen) and delivered in several types of vectors (nanoparticles, liposomes, virosomes). Next generation vaccines currently explored use immunization with tumor-specific neoantigens, that promotes responses against mutated tumor-specific epitopes [122].

Administration of TAA is frequently accompanied by an adjuvant to mobilize and/or activate DCs (GM-CSF, Flt3L, TLR agonists). For example, GM-CSF can directly stimulate DC differentiation, activation, and migration. The T-VEC, an attenuated oncolytic strain of herpes simplex virus engineered to express human GM-CSF and approved by the FDA, favored anti-tumor responses and increased survival in patients with advanced melanoma [123].

The TLR agonists (the most widely used being TLR3/7/8/9) induce the maturation of DCs with the increase in the expression of co-stimulation molecules and the reduction of their phagocytic capacity, together with the production of IL-12 and IFNα, thus promoting the Th1 polarization of T-cells [31,124]. The effectiveness of these treatments is currently the subject of numerous clinical trials in cancer patients.

The use of a TLR3 agonist (PolyI:C) showed high therapeutic potential in a pre-clinical mouse B16 melanoma model [125]. The agonist PolyI:C activates DCs, induces pro-inflammatory cytokines, drives NK cell activation, Th1 immunity and anti-tumor CD8 T-cell responses culminating in therapeutic tumor suppression [126]. Due to toxicity issues, other TLR3 agonists derived from PolyI:C (poly-ICLC, PolyI:C12U) are also the subject of current cancer research [127]. Intratumoral administration of PolyI:C derivatives are currently being tested in the clinic [128].

In addition, many clinical trials (phase I-III) evaluate the activation of pDCs in the tumor using TLR7 and TLR9 agonists—which reactivate their production of type I IFN and cytotoxic molecules (e.g., TRAIL) [66]. A clinical trial with subcutaneous injection of TLR9 ligands (OGN) in patients with advanced melanoma shows activation of pDCs and partial clinical response or stabilization of disease in 25% of patients [129]. In addition, the use of imiquimod (IMQ, TLR7 agonist) in a humanized melanoma mouse model inhibited tumor progression following the mobilization of pDCs and the induction of their cytotoxic functions [53]. It is also possible to target LCs or dermal DCs using TLR3 or TLR2/4/6 agonists, respectively [124].

However, in a clinical setting, TLR agonists show significant interest when combined with other immune-targeted therapies (ICB, DC-based vaccination) [31]. Indeed, the combination of a TLR9 agonist (SD-101) with the anti-PD-L1 monoclonal antibody, pembrolizumab is associated with a better infiltration of DCs in the tumor and promising clinical results in melanoma [130].

In addition, CD40 or STING agonists can also be used to activate DCs and induce CTL responses [115,131]. Intratumoral administration of a CD40-targeting antibody in a mouse model induces an effective systemic cytotoxic response [132]. In addition, the use of CD40 agonists may decrease PD-1 expression by T-cells, reversing their PD-L1-induced exhausted phenotype and rending tumors more susceptible to anti-PD-1 immunotherapies [133]. The CD40 agonists are mainly used in combination with other therapeutic approaches delivering tumor antigens to act as an adjuvant facilitating the activation of DCs [115]. Furthermore, the production of type I IFN following the activation of the STING pathway potentiates the presentation of tumor antigens by DCs and their cross-presentation to CD8 T-lymphocytes [131].

Treatments blocking inhibitory pathways, such as STAT3 and IDO, are currently being studied in the clinic and may affect DCs activity [31]. Inhibition of STAT3 and MAPK may promote the differentiation of CD14^+^ cells into moDCs and this was associated with an increase in the infiltration of effector T-cells (with a Th1 profile) in melanoma [134]. Inhibition of IDO would also restore T-cell activation by DCs. Several clinical trials with STAT3 or IDO inhibitors suggest limited efficacy as monotherapy but combining them with immunotherapies may be of clinical interest [31].

Insights into the metabolic suppression of melanoma-associated DCs allows designing therapeutic strategies based on metabolic interventions to promote/restore DC function [135]. For this purpose, an antagonist of the retinoic acid receptor α has been used to enhance the efficacy of peptide-pulsed DC vaccine, as it enhanced IL-12 production while dampened IL-10 and TGFβ secretion [136]. Neutralization of the adenosine signaling in DCs through pharmacological antagonists of the A_2B_ receptor or interference with the CD73 ectonucleotidase have been shown to improve the immunogenicity of DCs by preventing the deleterious immunosuppressive effect of adenosine on DC function and anti-tumor immunity [137,138]. The glycolytic and fatty acid metabolism can also be targeted to enhance the immunostimulatory capacities of DCs. In melanoma models, silencing the GLUT1 glucose transporter in melanoma cells had direct anti-tumor effects, but also enhanced DC-mediated anti-tumor immunity. Regulation of lipid levels in DCs by blocking fatty acid synthesis—by inhibiting acetyl-CoA carboxylase—improved anti-tumor immunity [139]. Thus, metabolic interventions can shift the profile of tumor-associated DCs from tolerogenic to immunogenic, supporting great promise for metabolism-based therapies to reinvigorate DCs.

### 5.2. Targeting DC Subsets In Vivo

It is relevant to use different adjuvants in combination with vectors specifically targeting DC to avoid potential adverse effects, which may occur with the recognition of these adjuvants by other immune cells. Some CLRs (mostly the endocytic CLRs) are specifically expressed by DC subtypes. These receptors have been used in several studies to specifically target particular DC subsets, and thus, to reduce adverse effects potentially induced by the activation of other immune cells [140]. Such direct targeting of DCs in vivo allows the harnessing of specialized DC subsets in their natural environment. The cDC1s are mostly targeted through DEC-205/CD205 and Clec9α, and cDC2s and MoDCs using DCIR, Dectin1, MR/CD206 or DC-SIGN. The candidate tumor antigens are thus coupled with antibodies or carbohydrates targeting a given CLR. Alternatively, these antigens can be enclosed into a vector (liposome, nanoparticles) coated with antibodies or carbohydrates targeting a CLR. This increases the probability of targeting a DC subset that in turn exerts antigenic presentation and activates T-lymphocytes.

Indeed, the fusion of an anti-DEC-205 antibody with a tumor antigen (MAGE-A3 or NY-ESO-1) induces a better activation of the CTL response compared to the tumor antigen alone (administered by electroporation of RNA or by pulse of peptides) [141,142]. As the targeting of myeloid DCs by DEC-205 does not induce their functions, the difference observed on the activation of the adaptive response would only be due to a better targeting of mature DCs by DEC-205 (presentation 100 times more effective) compared to the administration of the tumor antigen alone [141]. A phase I clinical trial evaluated the use of a vaccine named CDX-1401 in patients with advanced melanomas. This vaccine consists of the fusion of a specific anti-DEC-205 antibody and the NY-ESO-1 antigen coupled with different adjuvants (R848, poly-ICLC). Treatment with CDX-1401 induced an antigen-specific T-cell response in 60% of patients, and a stabilization or regression of the pathology in respectively 33 or 9.5% of patients [143]. Interestingly, 67% of patients (4 out of 6) who received anti-CTLA-4 therapy after their treatment with CDX-1401 responded to this second therapy, compared to 11% patients who received only anti-CTLA-4 treatment. In a phase II clinical trial in melanoma patients, the use of Flt3L in combination with CDX-1404 enhanced the proportions of the three subtypes of DCs in the blood. All the DC subsets are activated by the combination, and this allows the induction of humoral and cellular adaptive responses [144]. In addition, the targeted presentation of NY-ESO-1 to human DCs via an anti-CD206 antibody also results in the activation of a specific CTL response, absent during administration of the antigen alone [142].

It is also possible to specifically target cDC1s using an antibody directed against Clec9α. The administration of antigen coupled to an anti-Clec9α antibody in a mouse model resulted in the antigenic presentation of cDC1s but induced Treg, whereas accompanied by an adjuvant, this targeting of cDC1s promoted immune responses [145]. In addition, the administration of a tumor antigen coupled to an anti-Clec9α in a mouse model of melanoma allowed the selective cross-presentation of this antigen by cDC1s. When combined with adjuvants, it was possible to induce CTL responses and promote tumor eradication [146]. Moreover, depending on the adjuvant used, the polarization of the induced CD4 T-cells was not the same (i.e., polyI:C and curdlane induce Th1 or Th17, respectively). The targeting of cDC1s by Clec9α would then need to be combined with polyI:C in order to avoid tolerance, but also to promote anti-tumor cytotoxic responses [145]. In addition, the targeting of cDC1s, via a vector composed of the antigen coupled to a peptide with a high affinity for Clec9α (CBP-12; only 12 amino acids), makes it possible to induce a strong cytotoxic response in a mouse model of melanoma (responders or not to anti-PD-1) even in the absence of adjuvants [147]. It is also possible to deliver melanoma tumor antigens (MelA, GP100) specifically to cDC1s by using poly(lactic-co-glycolic acid) (PLGA) nanoparticles coated with the anti-Clec9α antibody [11] eventually in combination with an NKT (α-galactosylceramide) agonist [148]. These nanoparticles induce CD8 T-cell priming and effective anti-tumor responses both in a mouse model and in vitro where the activation of human cDC1s drives the expansion of antigen-specific CD8 T-cells.

It is also possible to target CLRs using their natural ligands, carbohydrates, rather than antibodies, although this strategy appears to be less effective in inducing adaptive responses (at least in the case of DC-SIGN) [149]. Indeed, targeting cDC2s via an antigen coupled to an anti-DC-SIGN antibody or glycans (Le^B^), in a B16-OVA mouse model with DCs expressing the human DC-SIGN, induces the priming of antigen-specific cytotoxic T-cells, and tumor regression is observed if the treatment is combined with Treg depletion [150]. Nanoparticles coated with a DC-SIGN ligand (gp120), encapsulating tumor antigens (gp100) and combined with adjuvants (R848, polyI:C), are less uptaken by human moDCs and induce weaker CD4 T-cell responses compared to nanoparticles coated with anti-DC-SIGN antibodies (even if the action of cytotoxic T-cells is equivalent between the two strategies). In addition, glycan-modified apoptotic melanoma-derived extracellular vesicles (ApoEVs) harboring DC-SIGN ligands allow the targeting moDCs and efficiently prime anti-tumor CD8 T-cells [151]. An efficient targeting of DCs through DC-SIGN using a trifunctional vaccine composed of mannosides conjugated to gp100 antigen and a TLR7 agonist favors antigen cross-presentation [152]. Thus, glycans/CLRs, crucially positioned at the interface between tumor cells and DCs, are emerging as promising immune checkpoints to exploit in order to reshape potent anti-tumor immunity.

### 5.3. DC Vaccines

Dendritic cell vaccines are developed using autologous PBMCs derived from patients, from which DCs or monocytes are purified, in order to produce moDCs ex vivo. These DCs are then pulsed with tumor antigens, matured with different stimuli, and reinjected into the patients. Following the use of different doses, distinct antigen loading strategies, routes of administration and activation methods, these treatments have fewer side effects than other immunotherapies, yet their clinical benefit remains limited (with average response rates of less than 15%) [153]. Recent reviews focusing on DC-based cancer vaccines in melanoma already fully detailed all clinical studies and strategies performed in this field [93,154,155,156]. Strategies to improve the efficacy of DC-based immunotherapy for melanoma are in development [93]. Different types of nanoparticulate antigen delivery systems for ex vivo-generated DC-based vaccines against melanoma are under development, combining effective delivery of antigen and high adjuvanticity [157]. In view of the difficulty of recovering large quantities of circulating DCs in humans, the use of moDCs has been more exploited so far. However, the effect of moDCs in vaccination settings remains limited due to their reduced cross-presentation and limited migration capacities in lymph nodes compared to other DC subtypes. In addition, endogenous DCs appear to be essential for the activity of moDCs-based vaccines, which would rather serve as vectors transporting tumor antigens to endogenous DCs [158]. The poor clinical outcome could also be due to the use of tumor antigens overexpressed or present in other tissues (NY-ESO-1, MUC1, MAGEA3, MART1, HER2) and the persistence of an immunosuppressive environment that would prevent the activity of DCs once entering the TME.

Therefore, many studies seek to specifically use natural endogenous DCs as vectors for DC vaccines (rather than artificial moDCs; given their potential for T-cell priming) in combination with adjuvants to stimulate them (Poly:ICLC, Flt3L), while attempting to abolish tumor-induced immunosuppression by combining these approaches with existing therapies such as ICBs or inhibitors of immunosuppressive cells. Obtaining a sufficient number of circulating DCs for the development of DC vaccines is possible following their isolation using magnetic beads coated with antibodies. The use of this type of vaccine in patients (especially done with cDC2s or pDCs) is not toxic, well tolerated and induces antigen-specific responses in some patients [159]. It is also possible to produce DCs in vitro in large numbers (especially cDC2s and pDCs), from CD34^+^ precursors, capable of inducing a strong anti-tumor response in vitro, with activation of T-lymphocytes and NK cells by cDC2s and pDCs, respectively [160]. Due to the low proportions of circulating cDC1s, this subtype of DC has not yet been used as a vector for DC vaccines, despite their high potential to induce cytotoxic T-responses. However, it has been made possible to produce, from CD34^+^ precursors, large number of cDC1s capable of cross-presenting long peptides to CD8 T-cells and activating NK cells, paving the way for the use of cDC1s for future vaccine approaches [161].

In the context of melanoma, administration of autologous cDC2s (3–10 × 10^6^ DCs) pulsed with tumor antigens (gp100, tyrosinase) in stage IV patients is non-toxic, well tolerated and induces the production of antigen-specific CD8^+^ T-cells in 30% of treated patients, 75% of whom survive without relapse for more than 15 months after the end of treatment [162]. However, there is an expansion of a population of BDCA1^+^CD14^+^ cells (possibly corresponding to DC3s) expressing PD-L1 in melanoma patients, which can be found in DC vaccines, and responsible for suppressing CD4 T-cell responses [92].

The administration of autologous pDCs, activated and pulsed with tumor antigens (gp100, tyrosinase), induces a systemic type I IFN response accompanied by the migration of pDCs in draining lymph nodes and triggering of antigen-specific CD4 and CD8 T-cells in patients with metastatic melanoma [55]. In addition, the median survival of these patients was 22 months compared to 7.6 months for patients treated with chemotherapy. Two years after the study, 47% of vaccinated patients were still alive (compared to 8% for patients treated with chemotherapy).

To date, no study allows prioritizing the use of autologous cDC2s or pDCs, according to their clinical benefits, for vaccination approaches in melanoma settings. However, vaccination with autologous pDCs, in patients with melanoma results in higher levels of chemokines attracting cytotoxic T-lymphocytes (CD8 T-cells, or γδ T-cells) compared to that with cDC2s (although a response is also observed with this strategy) [163]. It may therefore also be interesting to use the two DC subtypes combined in a vaccine setting in order to have a better subsequent response. Indeed, in a tumor mouse model, the combined administration of pulsed pDCs and cDC2s with OVA increased the amounts of OVA-specific CD8 T-cells compared to immunization with only one of the two subtypes of DCs [164]. In addition, pDCs seem necessary to potentiate the cross-presentation of cDC2s (dependent on their MHC-I expression).

It is also possible to use semi-allogeneic pDCs (restriction by HLA-A*0201), pulsed with tumor antigens, to induce an effective cytotoxic T-cell response in the context of melanoma in an in vivo model of humanized mice [165] or in vitro from patients’ PBMCs or tumor-infiltrating cells [166]. A phase Ib clinical trial, conducted on nine patients with stage IV melanoma who received this semi-allogeneic vaccine, shows treatment tolerance and increased tumor antigen-specific T-cells in 22% of patients, as well as stabilization of pathology in 44% of patients [167].

In addition, different methods have been developed to improve the intrinsic capabilities of DCs or to make them less sensitive to TME. A sialidase treatment of DCs, in order to remove their sialylated groups, before vaccination, improved their efficacy and the priming of antigen-specific T-cells presented in vitro in humans and in vivo in a mouse model [168]. It would also be possible to pretreat pulsed DCs with mRNAs (encoding TAAs) or siRNAs (targeting PD-L1 and PD-L2) prior to their administration in order to increase their ability to present tumor antigens to T-cells and to mitigate the impact of the TME on DCs in melanoma settings [169,170,171].

The therapeutic potential of DC vaccination may likewise be strengthened by immune checkpoint blockade. Indeed, some studies in the context of melanoma show an interest in combining DC vaccines with immunotherapies in order to improve the response to treatment and achieve a better clinical response [172,173]. The rationale of combining DC vaccines with immunomodulatory drugs is strongly sustained by the study of Bulgarelli et al. that revealed an increase in CD8^+^ TIL following vaccination with autologous DCs loaded with tumor lysates, thus demonstrating that DC vaccine sustains a T-cell inflamed TME [174]. A phase I/IIa clinical trial (NCT02678741) is currently exploring the effect of TLPLDC vaccine (i.e., activated autologous DCs that have phagocytized particles containing autologous tumor lysate) on the response to ICB treatment in patients with metastatic melanoma. Another phase I clinical trial (NCT03092453) is testing the efficacy and toxicity of combining a DC vaccine with anti-PD-1 therapy in patients with stage III-IV melanoma.

Strikingly, DCs drive circadian anti-tumor responses. Indeed, the rhythmic trafficking of DCs to the melanoma tumor draining lymph node governs a circadian response of tumor antigen-specific CD8^+^ T-cells that is dependent on the circadian expression of the co-stimulatory molecule CD80 [175]. Thus, the circadian rhythms of anti-tumor immune components are critical for optimal responses, and time of vaccination should be carefully adjusted, driving the field toward chrono-therapeutic vaccination.

Hundreds of clinical trials using DCs as targets or vectors, alone or in combination with adoptive cell or ICB therapies, have been carried out or are currently underway across the world in patients with advanced melanoma. Recent insights into DC subset biology and understanding of the regulation of DC function with the TME will probably translate soon into novel therapeutic strategies, in order to exploit the outstanding potential of these cells to restore efficient anti-tumor responses and achieve clinical successes.

## 6. Conclusions

Dendritic cells are outstanding cells with robust potentialities, crucially positioned at the center of anti-tumor immunity. Insights from recent years provide improved knowledge of the specialized functions of DC subsets, and of the regulation of DCs features within the TME. Such advances improve our understanding of the role of DCs during melanoma development and allow their therapeutic exploitation and the design of new therapeutic strategies.

As DCs shape both tumor development and anti-tumor immunity, these cells represent attractive targets to manipulate and exploit for clinical translations. The DCs serve as direct targets/vectors for therapy, but are also critical to shape the quality of therapy-associated anti-tumor immunity such as ICB or adoptive cell therapies. Harnessing the anti-tumor potential of DCs for cancer immunotherapy is required to overcome tumor-induced hijacking and immunosuppressive networks. Understanding mechanisms of tumor-induced DC subversion is critical to optimize current therapies and design future efficient immunotherapies for melanoma.

There are still issues and remaining challenges that will need to be addressed in future studies for next generation approaches. We need to better understand the pathophysiology of the different subtypes of DCs in melanoma, especially the newly discovered subsets mregDCs and DC3s, to better exploit their potentialities for therapies. Further insights into DCs’ diversity, features, spatial positioning, networking, regulation and shaping by the TME could lead to improved cancer therapies. A deeper exploration of the complex interplay between melanoma tumor cells and DCs, and of the subversion pathways is mandatory to design novel effective therapeutic options, in order to overcome the metabolically hostile tumor microenvironment, and exploit the gut microbiome to potentiate DC functions.

Altogether, due to their exceptional potential, DCs deserve to be positioned in the current melanoma immunotherapeutic landscape as part of combinatorial regimens together with adoptive cell and ICB therapies. Recent discoveries strongly motivate the pursuit of exploitation of the potential of DCs to drive robust anti-tumor immunity, offering promising tracks to achieve clinical successes. Altogether, DCs hold great promise to participate in achieving better patient clinical outcomes in the future.

Dendritic cells are central regulators of anti-melanoma immune responses and subsequent outcome. They display a puzzling role in the control of tumor development as they harbor both anti-tumor and pro-tumor functions. On one hand (left part, Figure 1), DCs are active players in orchestrating robust anti-tumor immunity by triggering cytotoxic CD8+ T-cells through TAA cross-presentation, recruiting and activating NK cells, ending up in tumor control. On the other hand (right part, Figure 1), melanoma tumor cells exploit DC versatility to subvert their functions. Many factors secreted by melanoma tumor cells interfere with DC differentiation, maturation, and function, leading to immunosuppressive/tolerogenic DCs and driving regulatory and Th2 responses. Abnormal glycans, unfavorable metabolic environment and specific microbiome can also negatively impact DC subsets. All these pathways favor tumor progression. This figure illustrates both the intra-tumoral heterogeneity of the DC subsets encountered in melanoma, the potential crosstalk between these DC subsets, and the prognostic value of DC subsets in melanoma.

Current therapies such as chemotherapies, immune checkpoint blockers and targeted therapies deployed in melanoma can indirectly positively affect DCs’ recruitment, differentiation, maturation, and function. Furthermore, insights into the mechanisms whereby melanoma hijacks the DCs has led to the design of therapeutic strategies that exploit the potential of DCs while preventing their subversion by the tumor. These strategies include approaches that mobilize, activate, energize and feed endogenous DCs, interventions that target specific DC subsets in vivo using TAA coupled to anti-CLR Abs or glycans, and DC vaccines.

## Figures and Tables

**Table 1:** Characteristics and functions of human DC subsets in the steady state or in an inflammatory setting [5,24,30,31].

DC Subset	Transcription Factors or Inducers	Markers	Main Functions	Ref.
cDC1s	ID2, IRF8, BATF3	BDCA3/CD141, CD11c^int^, DNGR1/CLEC9A, XCR1, CADM1, BTLA, CD26	CD8 T-cell activation Type III IFN secretion	[5,9,10,11]
cDC2s	ID2, ZEB2, IRF4, Notch2	BDCA1/CD1c, CD11c^hi^, CD11b, CD5, FCεR1, SIRPA/CD172a, CCR2, BTLA CLEC10A^+^ (cDC2B ^1^) or CLEC10A^−^ (cDC2A)	CD4 T-cell activation IL-12 secretion	[5,12,25]
pDCs	E2-2, ZEB2, IRF8, IRF4, IRF7	BDCA2/CD303, CD123, BDCA4/CD304, FCεR1, ILT3, ILT7	Antiviral immunity Type I IFN secretion	[14]
LCs	ID2, RUNX3	Langerin, CD1a, EpCAM, TROP2, E-cadherin	Induction of Th2 T-cells IL-15 secretion	[18,19]
DC3s	GM-CSF	BDCA1, CD163, CD11c, CD14^lo to hi^, MGL/CLEC10A, CD36, FCεR1	T-cell activation IL-12 & IL-23 secretion	[24]
moDCs/InfDCs	MAFB, KLF4 IRF8	BDCA1, CD14, CD11c, MR/CD206, CD1a, DC-SIGN, SIRPA	Inflammation (IL-1β, IL-6, IL-23, TNF-α)	[3]
mregDCs	C/EBPα	LAMP3/DC-LAMP, CD11b, XCR1, CD11c, CD103, CCR7^hi^	Tumor control (IL-12) Immune regulation (PD-L1, PD-L2)	[5,29]

^1^ this cDC2B subset may correspond to DC3s; Ref. means references.
